# Supplementary material for: Defining and measuring microservice granularity—a literature overview
Source: PeerJ Comput Sci. 2021 Sep 8;7:e695. doi: 10.7717/peerj-cs.695 (PMC8444086; doi:10.7717/peerj-cs.695)
Supplement: Supplemental Information 1 [file peerj-cs-07-695-s001.docx]

# **Appendix A.** Granularity approaches - tabulated data

| **Paper** | **Metrics** | **Phase** | **Approach** | **Research Strategies** | **Quality Attribute** | **Contribution** | **Experiment** | **Technique used** | **Input data** | **Conference - Journal** | **Case study** | **Automatic level** |
| --- | --- | --- | --- | --- | --- | --- | --- | --- | --- | --- | --- | --- |
| P1.   Microservices and their design trade-offs: A self-adaptive roadmap (Hassan and Bahsoon, 2016) | None | Design, runtime | Structural and behavioral aspects. | Solution proposal | No reports | Problem formulation | No reports | Self-adaptive solution in a MAPE-K cycle (Monitoring, analize, plan, execute, knowlegde) | None | IEEE Int. Conf. Serv. Comput. | None | Manual |
| P2.    Microservice Architectures for Scalability, Agility and Reliability in E-Commerce (Hasselbring and Steinacker, 2017) | None | Desing, development, deployment, test, migration | Structural and behavioral aspects. | Experience paper | Scalability, performance, and fault tolerance | Reference architecture | Case study | Vertical decomposition in self-contained systems. Automation is a key to define microservices granularty. | None | IEEE International Conference on Software Architecture Workshops, ICSAW 2017 | Real-life case study. Otto.de. | Manual |
| P3.    From Monolith to Microservices: Lessons Learned on an Industrial Migration to a Web Oriented Architecture (Gouigoux and Tamzalit, 2017) | Costs of Quality Assurance and the cost of deployment | Design, Migration | Structural | Solution proposal | Maintainability | Method | Case study | Balance between the costs of quality assurance and the cost of deployment. Try and error. | None | IEEE International Conference on Software Architecture Workshops, ICSAW 2017 | Real-life case study. MGDIS. http://www.mgdis.fr | Manual |
| P4.    Microservices: Granularity vs. Performance. (Shadija et al., 2017) | Response time, number of calls | Deployment, migration | Structural | Validation research | Perfonrmance: Latency | Evaluation or comparison | Case study | They compare same microservices in a single container and in two containers. | None | The 10th International Conference on Utility and Cloud Computing. ACM | In lab case study.  University admissions systems.  Central Admission authority, UK.  Simulated Deployment | Manual |
| P5.    Microservice Ambients: An Architectural Meta-Modelling Approach for Microservice Granularity. (Hassan et al., 2017) | None | Design, runtime | Structural and behavioral aspects. | Evaluation research | No reports | Reference architecture, ADL | Case study | Microservice ambientes as an Architecture Definition Language (ADL). Extends aspect -oriented architecture | None | International Conference on Software Architecture | Hypothetical case study.  Online movie streaming subscription-based system. | Manual |
| P6.    Microservices identification through interface analysis. (Baresi et al., 2017) | None | Design | Structural | Validation research | No reports | Method | Experiment, case study | Analysis of semantic similarity of the functionalities described in OpenApi. DISCO. Clustering: K-means | OpenApi specification, scheme.org | Lecture Notes in Computer Science. Springer International Publishing | Hypothetical case study.  Cargo Tracking.  Money Transfer  Kanban Board.   Real-life case study.  Real-world OpenAPI specification, from Apis.guru https://apis.guru/openapi-directory/ real-life case study | Automatic |
| P7.    Partitioning microservices: A domain engineering approach (Josélyne et al., 2018) | None | Design | Structural | Solution proposal | No reports | Method | Case study | Domain engineering, domain driven design | None | Proceedings of the 2018 International Conference on Software Engineering in Africa - SEiA ’18. ACM | Hypothetical case study.  Weather case study. | Manual |
| P8.    A Case Study on Measuring the Size of Microservices (Vural et al., 2018) | COSMIC function points | Design | Structural | Solution proposal | No reports | Method | Case study | Domain driven Design (DDD). COSMIC function points. | None | International Conference on Computational Science and Its Applications - ICCSA 2017. Springer | Hypothetical case study.  EShop | Manual |
| P9.    Identifying Microservices Using Functional Decomposition (Tyszberowicz et al., 2018) | None | Design | Structural | Validation research | No reports | Method | Experiment, case study | Functional decomposition | Use cases | International Symposium on Dependable Software Engineering: Theories, Tools, and Applications, Lecture Notes in Computer Science. Springer International Publishing | Hypothetical case study. CoCoME case study. | Manual |
| P10.  Unsupervised learning approach for web application auto-decomposition into microservices (Abdullah et al., 2019) | Response time, number of calls, the number of rejected requests | Migration, development, deployment | Structural and behavioral aspects | Validation research | Scalability, Performance | Method | Experiment, case study | Machine learning method, Scale Weighted K-means | Access logs | Journal of System and Software. | Hypothetical case study.  Opens source case study: Open source benchmark web application ACME Air. https://github.com/blueperf, https://github.com/blueperf/acmeair-driver | Automatic |
| P11. Requirements Reconciliation for Scalable and Secure Microservice (De)composition (Ahmadvand and Ibrahim, 2016) | Dependency weight. Security Impact (high, medium, low). Scalability (high, medium, low) | Design | Structural and behavioral aspects | Solution proposal | Scalability, sucurity | Methodology | Case study | Methodology for microservice decomposition from system requirements, considering Security and scalability | Use cases | IEEE 24th International Requirements Engineering Conference Workshops (REW) | Hypothetical case study.  Movie/tv shows streaming system. | Manual |
| P12. Function-Splitting Heuristics for Discovery of Microservices in Enterprise Systems. (De Alwis et al., 2018) | No. of requests, Ex. time (ms) Avg. memory (GB) Avg. disk (GB), Scalability, availability, and efficiency, Lack of cohesion Structural coupling | Development, deployment, migration | Structural and behavioral aspects | Validation research | scalability, availability, execution efficiency | Method | Case study | Heuristics used for functional splitting enterprise system to microservices. Microservice Discovery Algorithms. | Source code, database, execution call graphs | International Conference on Service-Oriented Computing - ICSOC 2018 | Real-life case study.  Open source projects. SUGAR and Church CRM. https://www.sugarcrm.com/. http://churchcrm.io/. | Automatic |
| P13. Extraction of Microservices from Monolithic Software Architectures. (Mazlami et al., 2017) | commit count (h), contributor count (c) and code size in LOC (s). Logical coupling (LC), contributor coupling (CC) AND semantic coupling (SC). team size reduction ratio metric (tsr). average domain redundancy metric | Development, migration | Structural and behavioral aspects | Validation research | performance and quality | Method | Experiment | Graph-based clustering algorithm. Class-based extraction model | Source code | IEEE International Conference on Web Services (ICWS) 2017 | Real-life case study.  Open source projects. Repositories using the Git VCS. (22 projects) | Automatic |
| P14. From Monolith to Microservices: A Dataflow-Driven Approach. (Chen et al., 2017)  P14. A dataflow-driven approach to identifying microservices from monolithic applications. (Shanshan Li et al, 2019) | None   Essential coupling and cohesion metrics Afferent Coupling (Ca), Efferent Coupling (Ce), Instability (I), and Relational Cohesion (RC), | Design, migration | Behavioral aspects | Validation research | No reports | Methodology | Case study Experiment | Top-down analysis approach - dataflow-driven decomposition algorithm. | Data flow diagram  Use Case | IEEE 24th Asia-Pacific Software Engineering Conference (APSEC).   Journal of System and Software | Hypothetical case study.  Movies and coments.  Cinema, movies, plan screening, online box, office list. | Manual  Semi-automatic |
| P15. From Monolithic Systems to Microservices: A Decomposition Framework based on Process Mining. (Taibi and Syst, 2019) | Coupling, Number of classes per microservices, Number of classes that need to be duplicated. | Development, migration | Structural and behavioral aspects | Validation research | No reports | Method | Case study | Process-mining approach, process-mining tool (DISCO or similar) is used to identify the business processes. | Execution Logs | Int. Conf. Cloud Comput. Serv. Sci. - CLOSER 2019 | Real-life case study  The company develops a document management system for bookkeeping, for Italian tax accountants | Semi-automatic |
| P16. Service Candidate Identification from Monolithic Systems based on Execution Traces. (Jin et al., 2019) | Integrating Interface Number (IFN), Cohesion at Message Level (CHM), Cohesion at Domain Level (CHD). 2) Modularity Quality measure.  Internal Co-change Frequency (ICF), External Co-change Frequency (ECF), Ration ofECF to ICF (REI). | Design, development, migration | Structural and behavioral aspects | Evaluation research | maintainability, scalability, functionality, Evolvability. Modularity | Method | Experiment, case study | Search-based functional atom grouping algorithm. Modify a Non-dominated Sorting Genetic Algorithm-II | Execution Traces from logs | IEEE Transactions on Software Engineering | Hypothetical case study.  Jpetstore (https://github.com/mybatis/jpetstore-6)   Real-life case study.  https://github.com/bvn13/SpringBlog  https://github.com/b3log/solo  https://sourceforge.net/projects/jforum2  https://roller.apache.org https://www.agilefant.com http://platform.xwiki.org | Automatic |
| P17. The ENTICE Approach to Decompose Monolithic Services into Microservices. (G. Kecskemeti et al, 2016)  Towards a Methodology to Form Microservices from Monolithic Ones (G. Kecskemeti et al, 2017) | None | Deployment, migration | Structural | Solution proposal | No reports | Methodology | No reports | Virtual machine image synthesis and virtual machine image analysis. | None | 2016 International Conference on High Performance Computing and Simulation, HPCS 2016  Euro-Par 2016 Workshops. | None | Manual |
| P18. Refactoring Orchestrated Web Services into Microservices Using Decomposition Pattern. (Mathawee Tusjunt and Wiwat Vatanawood, 2018) | None | Design | Structural | Solution proposal | No reports | Methodology | No reports | Generating scenario statements, defining vocabularies and relationships, and defining business capabilities. We obtain the list of business capabilities for use in subsequent processes. We input the BPEL web service of the existing system and collect the vocabularies from XML tags. Lastly, we refactor those words to the list of microservices. Words relationships business capabilities and BPEL description. | Scenario statements, workflow, BPEL description. | 2018 IEEE 4th International Conference on Computer and Communications (ICCC) | Hypothetical case study.  Online shopping workflow. | Manual |
| P19. A logical architecture design method for microservices architectures (Nuno Santos et al., 2019) | None | Design | Structural | Solution proposal | No reports | Method | Case study | UML use cases diagrams for domain modeling, which are further used as an input for designing a MSLA using a set of rule-based decisions, by using an adaptation of the Four Step Rule Set (4SRS) method. | UML use cases model | ECSA 2019 - ACM | Real-life case study  the Integrated Management Platform 4.0 (IMP_4.0), ERP System - | Manual |
| P20. A New Decomposition Method for Designing Microservices. (Al-Debagy, Omar, Martinek, Peter, 2019) | None | Design, migration | Structural | Validation research | No reports | Method | Case study | microservices decom- position method using word embedding and hierarchical clustering of semantic similarity method | OpenApi specification. | Journal Periodical Polytechnica Electrical Engineering and Computer Science. | Hypothetical case study.  Kanban board  Money transfe  Real-life case study  AWS - Real life PayPal - Reallife | Automatic |
| P21. Business Object Centric Microservices Patterns (De Alwis et al., 2019) | Structural Coupling, cohesion, lack of cohesion, no executions, packets sent, execution time, avg CPU (EC2, DB), Avg network | Desing, development, deployment, migration | Structural and Behavioral aspects | Validation research | Scalability, availability, performance | Patterns, Method | Case study, experiment | Microservice Discovery Algorithms. Gentic algorihtm, DISCO, Non-dominated Sorting Genetic Algorithm II (NSGA II) | Source code, database, execution call graphs | OTM Confederated International Conferences "On the Move to Meaningful Internet Systems" - 2019 | Real-life case study  Dolibarr - https://www.dolibarr.org/ Sugar- CRM. - https://www.sugarcrm.com/ | Semi-automatic |
| P22. From a Monolith to a Microservices Architecture: An Approach Based on Transactional Contexts. (L Nunes et al,, 2019) | Number of Singleton Clusters (NSC) Maximum Cluster Size (MCS) Silhouette Score. precision, recall and f-score | Design, development, migration | Behavioral aspects | Validation research | No reports | Methodology | Case study | Clustering algorithm is applied to aggregate domain entities that are shared by the same controllers | Source code, MVC architectural style.  Call graph. | 13th European Conference, ECSA 2019. | Real-life case study.  LdoD3 and Blended Workflow. https://github.com/socialsoftware/edition.  https://github.com/socialsoftware/blended-workflow.  https://ldod.uc.pt | Semi-automatic |
| P23. Granularity Cost Analysis for Function Block as a Service. (Aydin Homay et al., 2019) | maximum request time Request total time that used by request (route). Service composition cost. Service Decomposition Cost | Design | Behavioral aspects | Solution proposal | No reports | Problem formulation | No reports | Service granularity cost analysis- based method | None | IEEE International Conference on Industrial Informatics (INDIN) | None | Manual |
| P24. MicroValid: A Validation Framework for Automatically Decomposed Microservices. (Michel Cojocaru et al., 2019) | Granularity: Nanoentities, classes Coupling: Dependencies, strongly connected components Cohesion: nanoentities composition, relation composition, responsabilities composition, semantic similarity | Design, development, migration | Behavioral aspects | Validation research | No reports | Method | Experiment | None | None | Proceedings of the International Conference on Cloud Computing Technology and Science, CloudCom | Hypothetical case study.  Cargo Tracker Application. Booking System Trading System Movie Crawler System  Ticket Price Comparator | Manual |
| P25. Migration of Software Components to Microservices: Matching and Synthesis. (Christoforou, Andreas et al, 2019) | None | Design, migration | Structural aspects | Validation research | No reports | Methodology | Experiment | Ontology scheme search-based techniques and recommends the optimal synthesis of microservices yielded by Multi-Objective Genetic Algorithms | Component and microservices properties. | Proceedings of the 14th International Conference on Evaluation of Novel Approaches to Software Engineering | Hypothetical data for an experiment. | Semi-automatic |
| P26. Microservice Decomposition via Static and Dynamic Analysis of the Monolith. (Alexander Krause et al, 2020) | None | Desing, development, migration | Structural and behavioral aspects | Experience paper | No reports | Methodology | Case study | DDD, and refined the architectural design via dynamic software visualization to identify appropriate microservice candidates | None | Proceedings - 2020 IEEE International Conference on Software Architecture Companion, ICSA-C 2020 | real-word, legacy lottery application in\|FOCUS. real-life case study | Manual |
| P27. Towards Automated Microservices Extraction Using Muti-objective Evolutionary Search. (Islem Saidani et al., 2019) | Cohesion, coupling CoHesion at Message level CoHesion at Domain level OPeration Number InteRaction Number | Design, migration | Structural and behavioral aspects | Solution proposal | No reports | Method | Experiment | Non-dominated Sorting Genetic Algorithm II (NSGA-II) | Source code | 17th International Conference Service-Oriented Computing | Real-life case study  JPetstore. https://github.com/mybatis/jpetstore-6. Spring- blog - https://github.com/Raysmond/SpringBlog | Automatic |
| P28. Extracting Microservices’ Candidates from Monolithic Applications: Interface Analysis and Evaluation Metrics Approach. (Al-Debagy, Omar Martinek, Peter, 2020) | lack of cohesion metric “LCOM” Number of Operations | Design, development, migration | Structural | Solution proposal | No reports | Method | Case study | Operation names are converted into vector representations using fastText model. Clustering using Affinity Propagation algorithm.  clustering semantically similar API’s operations | OpenAPI specification file | 2020 IEEE 15th International Conference of System of Systems Engineering (SoSE) | Hypothetical case study. Kanban Boards Money Transfer | Automatic |
| P29. Migrating Web Applications from Monolithic Structure to Microservices Architecture. (Zhongshan Ren et al., 2018) | Coupling  Cohesion  PA(package analysis), SSA (static structure analysis), CHA (class hierarchy analysis), SCGA (static call graph analysis), and CSDA (combined static and dynamic analysis) | Design, development, migration | Structural and behavioral aspects | Validation research | Scalability and performance | Method | Experiment | function call graph Markov chain model to represent migration characteristics. Clustering method k-means hierarchical clustering method | Source code, runtime logs | ACM International Conference Proceeding Series | Real-life case study  DayTrader,  JPetstore,  TPC-W  RUBiS  12 applications migrated | Semi-automatic |
